# High Efficiency Organic/Silicon-Nanowire Hybrid Solar Cells: Significance of Strong Inversion Layer

**DOI:** 10.1038/srep17371

**Published:** 2015-11-27

**Authors:** Xuegong Yu, Xinlei Shen, Xinhui Mu, Jie Zhang, Baoquan Sun, Lingsheng Zeng, Lifei Yang, Yichao Wu, Hang He, Deren Yang

**Affiliations:** 1 State Key Laboratory of Silicon Materials and School of Materials Science and Engineering, Zhejiang University, Hangzhou 310027, China; 2 Institute of Functional Nano and Soft Materials (FUNSOM) & Collaborative Innovation Center of Suzhou Nano Science and Technology, Soochow University, Suzhou 215123, China

## Abstract

Organic/silicon nanowires (SiNWs) hybrid solar cells have recently been recognized as one of potentially low-cost candidates for photovoltaic application. Here, we have controllably prepared a series of uniform silicon nanowires (SiNWs) with various diameters on silicon substrate by metal-assisted chemical etching followed by thermal oxidization, and then fabricated the organic/SiNWs hybrid solar cells with poly(3,4-ethylenedioxythiophene): poly(styrenesulfonate) (PEDOT:PSS). It is found that the reflective index of SiNWs layer for sunlight depends on the filling ratio of SiNWs. Compared to the SiNWs with the lowest reflectivity (LR-SiNWs), the solar cell based on the SiNWs with low filling ratio (LF-SiNWs) has a higher open-circuit voltage and fill factor. The capacitance-voltage measurements have clarified that the built-in potential barrier at the LF-SiNWs/PEDOT:PSS interface is much larger than that at the LR-SiNWs/PEDOT one, which yields a strong inversion layer generating near the silicon surface. The formation of inversion layer can effectively suppress the carrier recombination, reducing the leakage current of solar cell, and meanwhile transfer the LF-SiNWs/PEDOT:PSS device into a p-n junction. As a result, a highest efficiency of 13.11% is achieved for the LF-SiNWs/PEDOT:PSS solar cell. These results pave a way to the fabrication of high efficiency organic/SiNWs hybrid solar cells.

Crystalline silicon (Si) solar cell has played an important role for many years in photovoltaic (PV) industries due to its excellent optical properties and high power conversion efficiency (PCE)^1^. However, the conventional fabrication process of crystalline silicon solar cell is quite complicated, which needs a high-temperature diffusion or expensive implantation process to form a p-n junction. Therefore, the cost of crystalline silicon solar cell based on p-n junctions is difficult to be reduced for wide application in the world. In order to reduce the cost, various technologies have been explored by simplifying the fabrication process at low temperatures^2^,^3^,^4^. Compared to p-n junction, Schottky junction has the merits of low-cost and easy fabrication, which is usually based on metal-silicon contact^5^,^6^. However, the thick metal layer in a conventional Schottky junction generally absorbs most of solar radiation, which strongly limits the improvement of solar cell efficiency. Recently, the silicon/poly(3,4-ethylenedioxythiophene):poly(styrenesulfonate)(PEDOT:PSS) hybrid solar cell which combines the advantages of organic and inorganic materials has been extensively studied^7^,^8^,^9^,^10^,^11^. At the beginning, the silicon/PEDOT:PSS solar cell is thought to work as a Schottky diode, in which the transparent PEDOT:PSS film functions as metal-like contact on n-type silicon wafer^12^. However, it has recently been recognized that the silicon/PEDOT:PSS solar cell is actually similar to a conventional p-n junction solar cell^13^. Usually, the efficiency of planar-Si/PEDOT:PSS solar cells can be above 10%^14^,^15^. By applying silicon nanowires (SiNWs) to reduce sunlight reflectivity, the performances of organic/silicon hybrid solar cells can be pushed to a new stage^16^,^17^,^18^. Recently, it has been reported that an efficiency up to 17.4% has been obtained for the PEDOT:PSS/silicon solar cell based on a back junction^19^. Furthermore, an efficiency of 13.6% was achieved for silicon/organic hybrid solar cells with the c-Si thickness of only about 20 μm by forming a front-side surface texturing of hierarchically bowl-like nanopores and a back surface field^20^. Nevertheless, high carrier surface recombination of SiNWs strongly restricts the improvement of solar cell performances. A better understanding on the trade-off between low light reflection and high carrier surface recombination of SiNWs is urgently needed for the organic/SiNWs solar cells.

Here, we have prepared a series of uniform SiNWs with different diameters for the fabrication of SiNWs/PEDOT:PSS solar cells. It is found that the reflective index of SiNWs layer for sunlight depends on the filling ratio of SiNWs. The solar cell based on the SiNWs with low filling ratio (LF-SiNWs) has a higher open-circuit voltage (*V*_*oc*_) and fill factor (*FF*) than the one based on the SiNWs with the lowest reflectivity (LR-SiNWs). It is clarified that the built-in potential barrier at the LF-SiNWs/PEDOT:PSS interface is much higher, which yields a strong inversion layer near the silicon surface. The formation of inversion layer cannot only suppress the carrier recombination and reduce the leakage current of solar cell, but also transfer the the device into a p-n junction. Therefore, the highest efficiency of 13.11% can be achieved for the LF-SiNWs/PEDOT:PSS solar cell.

## Experimental procedures

### SiNW Preparation

300 μm thick, n-type single-side polished 100-oriented crystalline silicon wafers with a resistivity of 0.1–0.3 Ω cm were used for experiments. The fabrication of uniform SiNWs was based on metal-assisted chemical etching (MACE) of silicon wafers by using Au metal mesh with nanoholes prepared from anodic aluminum oxide (AAO) membranes^21^. Firstly, Au was sputtered onto the surface of an AAO membrane to form metal mesh with 150–250 nm diameter nanoholes. The Au mesh was transferred to the silicon substrate after being released from the AAO membrane by floating in 0.1 M NaOH solution for 2 h. The SiNWs were grown by immersing the Au-coated silicon substrate into mixture of 4 M hydrofluoric (HF) acid solution (40 wt%) and 0.2 M H_2_O_2_ (30 wt%) solutions at room temperature. Finally, a series of SiNWs with different diameters were obtained by annealing the samples in dry oxygen thermal oxidation treatment at 900^°^C for different times, followed by the immersion in diluted HF acid solution (5  wt%) for 60 s.

### Device Fabrication

For the solar cell fabrication, the samples were first immersed in a tetramethylammonium hydroxide (TMAH) solution (1 wt%) to smooth the walls of SiNWs and then dipped in a HF solution to provide Si-H termination. Meanwhile, 5 wt% dimethyl sulfoxide (DMSO) and 1 wt% hexaethylene glycol monododecyl ether (Triton X-100) were added into the aqueous PEDOS:PSS dispersion (Clevios PH 1000) to improve the conductivity and adhesion of PEDOT:PSS on silicon. The improved PEDOT:PSS film was spin-coated on the SiNWs sample with a rate of 3000 r/min, and then annealed at 125 ^°^C for 7 min. Finally, silver (Ag) grids were deposited onto the PEDOT:PSS film as the front electrodes and the aluminum (Al) film as the back contact.

### SiNW and device characterization

The morphology and structure of SiNWs were characterized by a field-emission scanning electron microscope (FE-SEM) (HITACHI S4800 and SU70) and a transmission electron microscope (TEM) (Tecnai G2 F20S-TWIN, FEI). Optical reflectance spectra were measured with a UV–VIS–NIR spectrophotometer (HITACHI U-4100). The current density *versus* voltage (*J-V*) characteristics of the hybrid solar cells were measured with a Keithley 2400 source unit under a simulated air mass AM1.5 solar spectrum irradiation source at 100 mW/cm^2^, and a calibrated silicon solar cell as a reference. The EQE spectrum was recorded using a Stanford Research System Model SR830 Lock-in Amplier unit coupled with a monochromator and a 500 W xenon lamp, calibrated by a silicon photodiode with known spectral response.

## Results and Discussion

**Figure 1:**
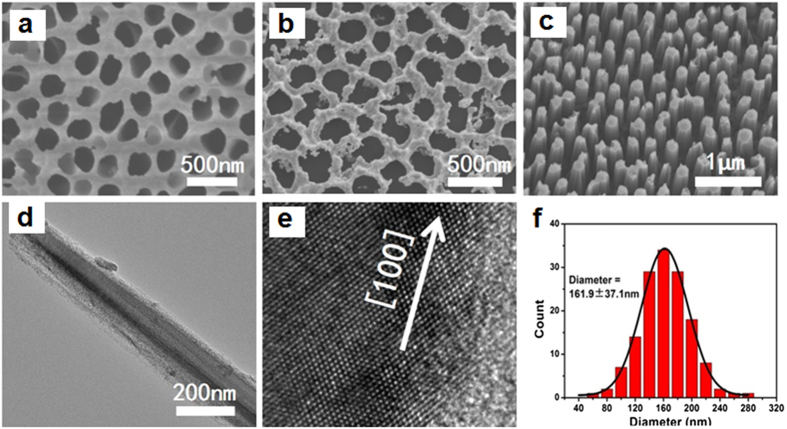
(**a,b**) SEM images of a Au-coated AAO membrane and a Au mesh-coated silicon wafer, respectively; (**c**) SEM image of SiNWs; (**d**) TEM diagram of an individual SiNW; (**e**) HRTEM diagram of a SiNW; (**f**) Histogram of the SiNWs diameter distribution, together with a Gaussian fit (solid line) of the measured statistical data.

Figure 1(a,b) show the SEM planar-images of an AAO membrane with Au coating formed by sputtering Au for 60 s and the Au mesh coated silicon substrate. It can be seen that the porous morphology of Au mesh is a good replication of the AAO membrane. The average pore diameter of Au-mesh is about 180 nm. After immersing the Au mesh-coated silicon substrate into an aqueous H_2_O_2_/HF solution, the SiNWs can be fabricated since the silicon parts under the Au mesh are etched off by Au catalyzation, as presented in Fig. 1(c). Figure 1(d) shows the TEM micrograph of an individual SiNW. It can be seen that the diameter of the SiNWs gradually increases from the top to the bottom. This phenomenon could be explained by the longer lateral etching of the SiNW upper part. Figure 1(e) shows the high resolution TEM (HRTEM) image of a SiNW. One can see that the axis of the nanowire is along the [100] crystallographic direction, consistent with that of the silicon wafer. To obtain the quantitative information on the SiNW sizes, the statistic distribution of the SiNW diameters is made. The result shows that the SiNW diameter fits a Gaussian-function distribution well, which is basically in agreement with that of the AAO nanoholes. If the diameter of a nanowire is presented by the middle of the nanowire, the mean and standard deviation of the SiNW diameter is 162 and 37 nm, seeing Fig. 1(f).

**Figure 2:**
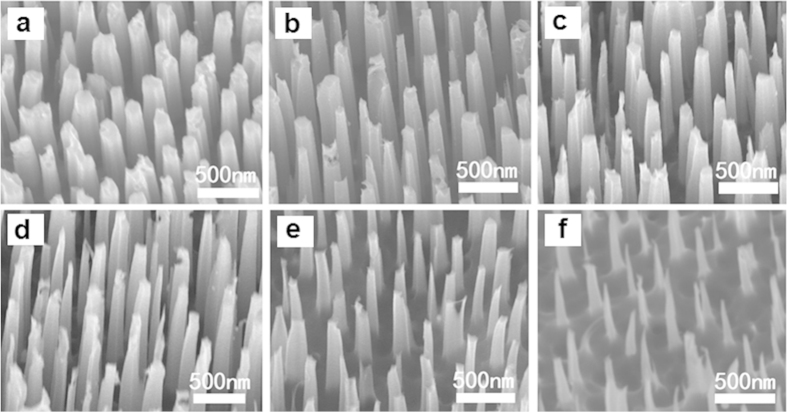
SEM images of the SiNWs obtained by varying the thermal oxidation time. (**a**) 0 min; (**b**) 45 min; (**c**) 90 min; (**d**) 180 min; (**c**) 300 min; (**d**) 600 min.

**Figure 3:**
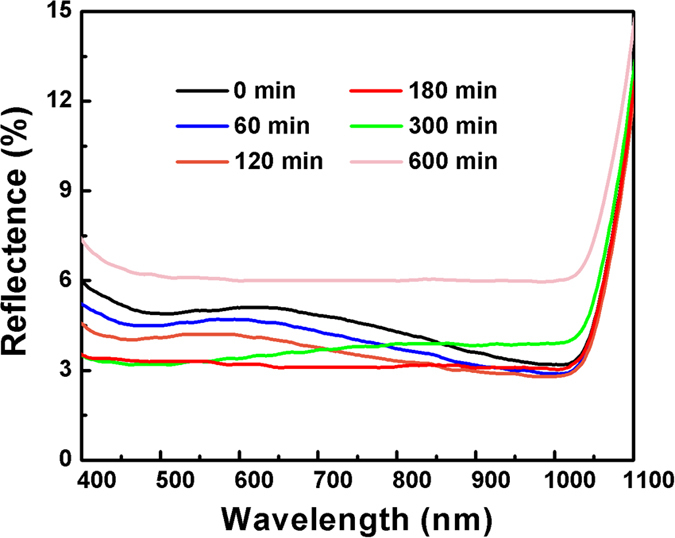
Reflectance of SiNWs subjected to thermal oxidation for different time followed by HF acid etching.

In order to obtain the SiNWs with different diameters, the as-grown SiNWs were treated by combining thermal oxidation and HF acid etching. Figure 2(a–f) present the SEM planar-images of SiNWs subjected to different oxidation time, followed by HF acid etching. It can be clearly seen that the diameter of SiNWs decreases with an increase of thermal oxidization time. Figure 3 shows the light reflectance of SiNWs with various diameters as a function of wavelength. It can be seen that the reflectivity of SiNWs in the entire light wavelength range gradually decreases with the filling ratio of SiNWs decreasing at the beginning, and reaches a minimum value in the case of 180 min thermal oxidization, which is ~3% in the nearly whole wavelength range. Afterwards, the reflectance in the long wavelength range starts to increase with an increase of thermal oxidization time. In the case of 600 min thermal oxidization, the reflectivity of SiNWs increases to ~6% in the entire light wavelength. It suggests that there exists an optimal diameter of SiNWs for the lowest reflectance. This can be explained by the variation in the reflective index of SiNWs layer if it is simply treated as an anti-reflective layer. The reflective index of SiNWs layer depends on the filling ratio of SiNWs. It is believed that in the case of 180 min thermal oxidization, the reflective index of SiNWs layer should be equal to the square root of silicon reflective index, about 1.8.

**Figure 4:**
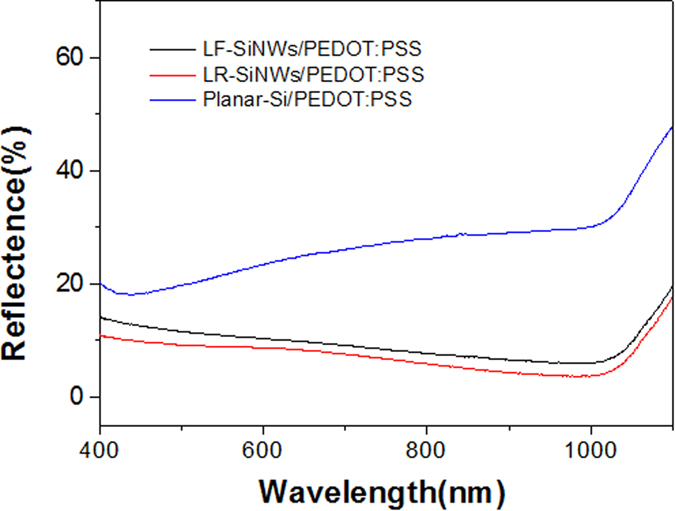
The reflectance of planar-Si/PEDOT:PSS, LF-SiNWs/PEDOT:PSS and LR-SiNWs/PEDOT:PSS samples.

**Figure 5:**
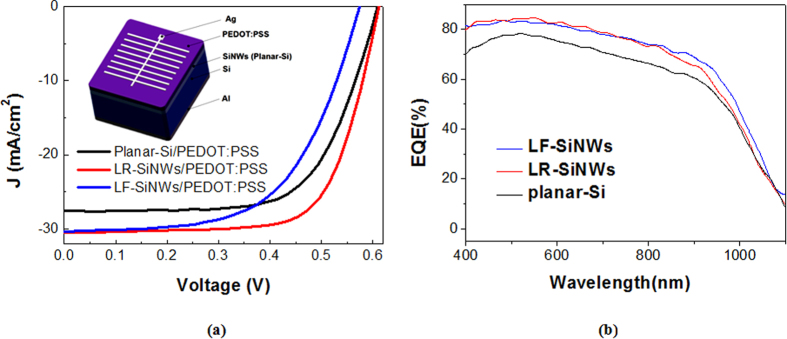
(**a**) Illuminated J-V characteristics and (**b**) EQEs of planar-Si/PEDOT:PSS, LF-SiNWs/PEDOT:PSS and LR-SiNWs/PEDOT: PSS solar cells.

**Figure 6:**
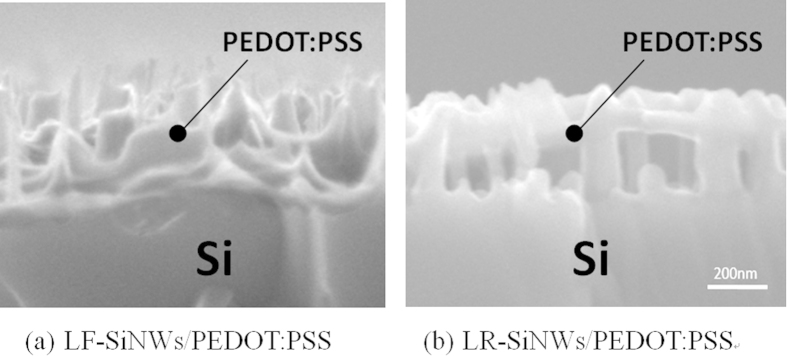
SEM cross-sectional micrographs of various SiNWs/PEDOT:PSS samples.

Here, we choose the typical SiNWs with the lowest reflectivity (labeled as LR-SiNWs) and the lowest filling ratio (labeled as LF-SiNWs) to fabricate the SiNWs/PEDOT:PSS solar cells, respectively. The reflectance of the samples with PEDOT:PSS coating is shown in Fig. 4. It can be seen that the reflectance of the samples can be changed by PEDOT:PSS coating. The PEDOT:PSS coating can increase the reflectance of both samples. It is believed that the coating or filling of PEODT:PSS film at the SiNWs can modify the reflective index of SiNWs layer and therefore degrade the reflectance of SiNWs. Figure 5(a) shows the *J-V* characteristics of various SiNWs/PEDOT:PSS solar cells, and the inset is the schematic diagram of our silicon/PEDOT:PSS hybrid solar cell. The corresponding electrical parameters are summarized in Table 1. Note that the *J-V* characteristic of the planar-Si/PEDOT:PSS solar cell is also shown as a reference. It can be seen that the LF-SiNWs/PEDOT:PSS and LR-SiNWs/PEDOT:PSS solar cells have a similar short-circuit current (*J*_*sc*_), much larger than the planar-Si/PEDOT:PSS one. The external quantum efficiencies (EQEs) of solar cells are shown in Fig. 5(b). It can be seen that the EQEs of LF-SiNWs/PEDOT:PSS solar cell is almost the same as that of LR-SiNWs/PEDOT:PSS one, much larger than that of planar-Si/PEDOT:PSS one. By integrating the EQE spectra, the short-circuit currents of the planar-Si/PEDOT:PSS, LF-SiNWs/PEDOT:PSS and LR-SiNWs/PEDOT:PSS solar cells are 27.6, 30.4, 30.3 mA/cm^2^, respectively, which are in good agreement with the illuminated I-V measurement results. The *V*_*oc*_ of the LF-SiNWs/PEDOT:PSS solar cell is much larger than that of the LR-SiNWs/PEDOT:PSS one, close to that of the planar-Si/PEDOT:PSS one. The reason will be described later. Compared to the planar-Si/PEDOT:PSS solar cell, the fill factor of the LR-SiNWs/PEDOT:PSS solar cell becomes much smaller, due to the increase of series resistance. Figure 6 compares the SEM cross-sectional micrographs of various SiNWs with PEDOT:PSS coating. It can be seen that the coverage of LF-SiNWs/PEDOT:PSS is much better than that of LR-SiNWs/PEDOT:PSS. This phenomenon must be associated with the difficulty of spin-coating of uniform PEDOT:PSS film on the thick SiNWs, as previous literature reported^22^. However, the fill factor of the LF-SiNWs/PEDOT:PSS solar cell is slightly larger than that of the planar-Si/PEDOT:PSS solar cell. It is believed that the spin-coating of PEDOT:PSS film should be more or less uniform on the LF-SiNWs, and meanwhile, the relatively larger area contact at the PEDOT:PSS/the LF-SiNWs decreases the contact resistance of hybrid solar cell. As a result, a maximum PCE of 13.11% is obtained for the LF-SiNWs/PEDOT:PSS solar cell, which is much larger than those of both LR-SiNWs/PEDOT:PSS solar cells and planar-Si/PEDOT:PSS.

**Figure 7:**
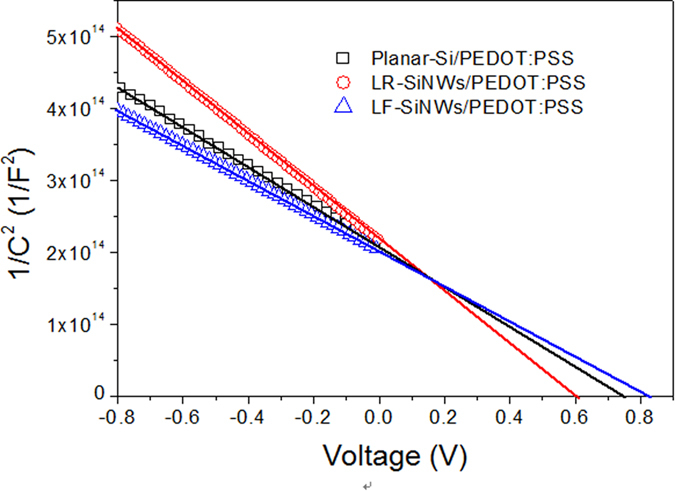
*1/C*^*2*^*-V* plots of the Planar-Si/PEDOT:PSS, LR-SiNWs/PEDOT:PSS and LF-SiNWs/PEDOT:PSS hybrid solar cells.

**Figure 8:**
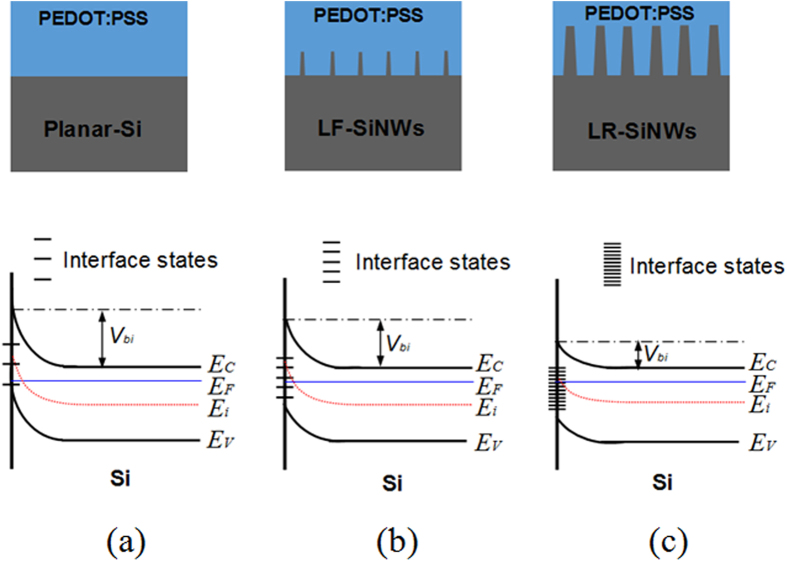
The density of interface states (DOS) and energy band diagram near the silicon surface in the (**a**) Planar-Si/PEDOT:PSS, (**b**) LR-SiNWs/PEDOT:PSS and (**c**) LF-SiNWs/PEDOT:PSS hybrid solar cells.

Figure 7 shows the capacitance variation of PEDOT:PSS/silicon devices with reverse bias voltage. According to Anderson’s model^23^, the capacitance of the device can be described as follows


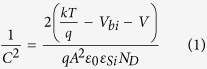


where *C* is capacitance, k the Boltzman constant; T the temperature; *V*_*bi*_ the built-in potential; *V* the applied voltage; q the unit charge; A the diode area; 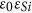 the permittivity of silicon and N_D_ the dopant concentration of silicon. The value of *V*_*bi*_ can be extracted from the extrapolation of the linear portion of the *1/C*^2^ *-V* plots, which are 0.80, 0.77 and 0.60 eV for the planar-Si/PEDOT:PSS, LF-SiNWs/PEDOT:PSS and LR-SiNWs/PEDOT:PSS solar cells, respectively. It shows that the *V*_*bi*_ of LF-SiNWs/PEDOT:PSS solar cell is slightly lower than that of Planar-Si/PEDOT:PSS one, but much larger than that of LR-SiNWs/PEDOT:PSS one. The higher *V*_*bi*_ of the LF-SiNWs/PEDOT:PSS and Planar-Si/PEDOT:PSS solar cells can significantly contribute to the improvement of *V*_*oc*_. The value of *V*_*bi*_ usually reflects the band bending degree near the silicon surface. If the *V*_*bi*_ is large enough, the band bending near the Si surface can cause the formation of an inversion layer^24^. The critical value of *V*_*bi*_ for generating a strong inversion layer can be described as follows,


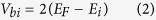


where *E*_*F*_ and *E*_*i*_ represent Fermi energy level and intrinsic Fermi energy level of Si sample, respectively. From the C-V measurement, it can be obtained that the dopant concentration in our silicon substrate is 9 × 10^15^/cm^3^. The critical value of *V*_*bi*_ for the formation of strong inversion layer is determined to be 0.76 eV. Therefore, we can judge that a strong inversion layer has been formed near the silicon surface for the LF-SiNWs/PEDOT:PSS solar cell as well as the planar-Si/PEDOT:PSS one, but it is not the case for the LR-SiNWs/PEDOT:PSS one. This result should be strongly associated with the density of electronic states (DOS) at silicon surface in different devices, seeing Fig. 8. The electronic states at the silicon surface generally originate from the silicon dangling bonds or other defects. If assuming that the surface states has the same energy distribution in the silicon band gap, the value of DOS should be proportional to the specific surface area of SiNWs, as we previously reported in ref. 25. Thus, it can be deduced that the LF-SiNWs have introduced lower DOS than the LR-SiNWs, but higher DOS than the Planar-Si. For the LR-SiNWs/PEDOT:PSS solar cell, the high DOS can pin the Fermi level at certain degree, and therefore the band bending become more difficult near the silicon surface. Since the LF-SiNWs/PEDOT:PSS solar cell has much low density of interface states, the band bending near the silicon surface is mainly dependent on the difference of Fermi levels between silicon and PEDOT:PSS, instead of the interface states. The formation of strong inversion layer can effectively reduce the electron population near the silicon surface, which benefits the decrease of minority carrier (hole) recombination rate at silicon/PEDOT:PSS interface.

**Figure 9:**
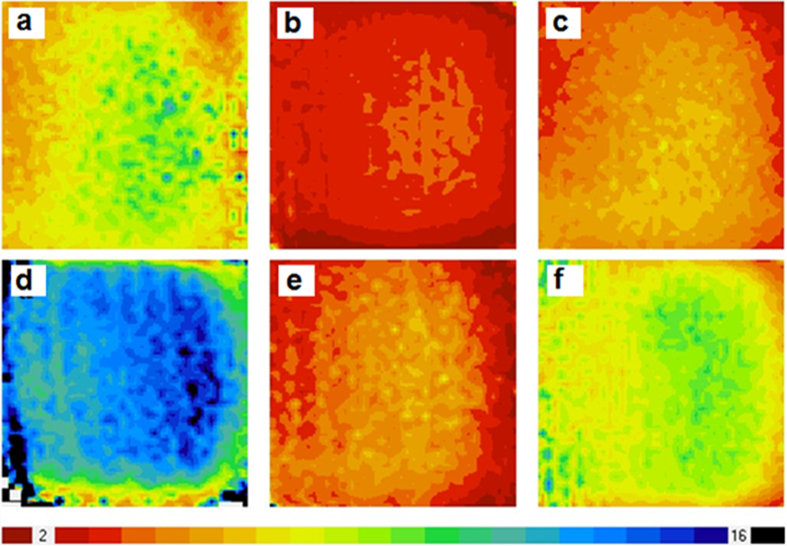
Mapping of the carrier lifetime of (**a**) planar-Si, (**b**) LR-SiNWs, (**c**) LF-SiNWs samples, and mapping of the carrier lifetime of (**d**) planar-Si, (**e**) LR-SiNWs, (**f**) LF-SiNWs samples after spin-coating of PEDOT:PSS films.

Figure 9 compares the minority carrier lifetime mappings of planar-Si, LR-SiNWs and LF-SiNWs samples before and after spin-coating of PEDOT:PSS film measured by microwave photo-conductance decay (MW-PCD) technique. Generally, the minority carrier lifetime of silicon samples depends on both the surface and bulk recombination as follows


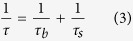


where *τ* is the measured carrier lifetime, *τ*_*b*_ the bulk carrier lifetime, *τ*_*s*_ the surface carrier lifetime. Since the bulk carrier lifetime of silicon wafer is much higher than the surface carrier lifetime, the measured carrier lifetime actually reflects the surface recombination lifetime of minority carriers. The measured carrier lifetime mappings of planar-Si, LR-SiNWs and LF-SiNWs show an average lifetime of 7.4, 3.3 and 5.1 μs, respectively. After spin-coated by PEDOT:PSS film, the carrier lifetimes of the three samples are improved at different level with the average lifetime of 12.3, 4.4 and 8.5 μs, respectively. It indicates that the PEDOT:PSS film can effectively passivate the silicon surface states. Note that the carrier lifetime of the LF-SiNWs sample get significantly improved, much larger than the LR-SiNWs. It is known that the carrier lifetime of sample is determined by the carrier recombination in the silicon bulk or/and silicon surface. Since the bulk hole lifetimes of our silicon wafer is larger than 100 μs, which is much higher than the measured effective hole lifetime, the surface recombination of holes actually dominates the effective carrier lifetime and therefore the short-circuit current. The surface recombination rate of holes at the silicon surface is determined by the DOS and the electron concentration at the silicon surface. As we previously report in ref. 25, the value of DOS at the LF-SiNWs sample surface should be smaller than that at the LR-SiNWs one. Meanwhile, the formation of strong inversion layer can significantly decrease the electron concentration near the silicon surface. Thus, the surface recombination rate of LF-SiNWs is much smaller than that of LR-SiNWs. The higher carrier lifetime in the LF-SiNWs sample can effectively trade off the negative influence of its low light reflectance on the *J*_*sc*_, compared to the LR-SiNWs one. Therefore, the LF-SiNWs/PEDOT:PSS solar cell almost has the same *J*_*sc*_ as the LR-SiNWs/PEDOT:PSS one.

**Figure 10:**
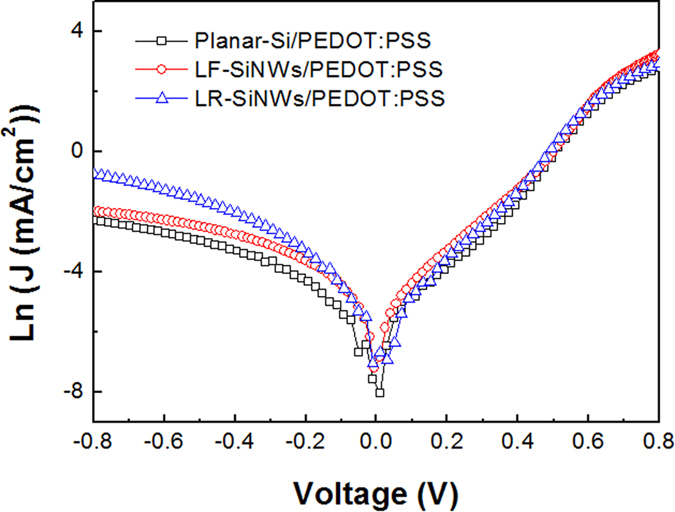
Current density versus voltage characteristic of the Planar-Si/PEDOT:PSS, LR-SiNWs/PEDOT:PSS and LF-SiNWs/PEDOT:PSS hybrid solar cells.

Figure 10 shows the *J-V* characteristics of solar cells in dark circumstance. The leakage current densities (*J*_*0*_) of a solar cell can be obtained according to the thermionic emission model^26^ as follows


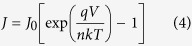


where *J* is the current density value, *V* is the applied voltage, *n* is the ideal factor, *T* is the absolute temperature (298 K), *k* is the Boltzmann constant (1.38 × 10^–23^ J/K), and q is the electronic charge (1.6 × 10^–19^ C). The values of *J*_*0*_ can be obtained to be 3.2 × 10^–8^, 3.8 × 10^–8^ and 8.1 × 10^–7^ A/cm^2^ for the planar-Si/PEDOT:PSS, LF-SiNWs/PEDOT:PSS and LR-SiNWs/PEDOT:PSS solar cells, respectively. One can notice that the *J*_*0*_ of the planar-Si/PEDOT:PSS and LF-SiNWs/PEDOT:PSS solar cells is one order of magnitude smaller than that of the LR-SiNWs/PEDOT:PSS one. The relationship between the *V*_*oc*_ and *J*_*0*_ can be expressed as follows


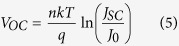


According to Eq. (5), it can be deduced that a smaller saturation current for the solar cell should be beneficial for a larger *V*_*oc*_. Therefore, the *V*_*oc*_ for both planar-Si/PEDOT:PSS and LF-SiNWs/PEDOT:PSS solar cells are larger than that for the LR-SiNWs/PEDOT:PSS one.

**Figure 11:**
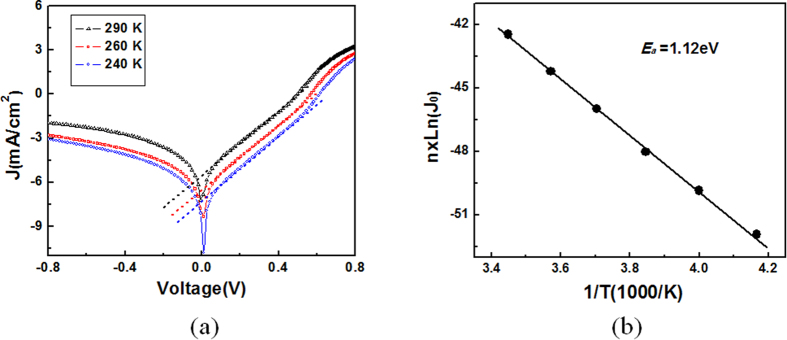
(**a**) The temperature dependent dark *I−V* characteristic and (**b**) *n* × ln(*J*_0_) versus 1/*T* characteristics of a LF-SiNWs/PEDOT:PSS solar cell yielding an activation energy barrier of ~1.12 eV.

Figure 11(a) shows the temperature dependent dark *I−V* characteristic of the LF-SiNWs/PEDOT:PSS solar cell. The dark *J−V* characteristic of solar cell can be further expressed as^27^,


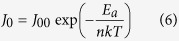


where *J*_*00*_ the temperature-independent factor and *E*_*a*_ thermal energy barrier responsible for current rectification. The formation of strong inversion layer should transfer the LF-SiNWs/PEDOT:PSS solar cell into a p-n homojunction. In the p-n junction model, the total dark-current density *J* constitutes the diffusion current density *J*_*d*_ (e.g. carrier diffusion in neutral regions) and the generation-recombination current density *J*_*r*_ (e.g. interface recombination). When the *J*_*r*_ is insignificant, the *J* is dominated by *J*_*d*_. In this case, the *J* is primarily determined by the intrinsic carrier concentration *n*_*i*_ of the silicon material. Since *J* ≈ *J*_*d*_ ∝ *n*_*i*_^2^∝ exp(-*E*_g_/*kT*), the thermal energy barrier responsible for current rectification should be equal to the silicon band gap energy^27^. According to Eq. (5), when qV is larger than kT, the ideality factor of device can be determined by fitting the plot of ln(*J*)-*V* in the linear regime as follows,


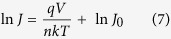


It is found that the ideality factor of LF-SiNWs/PEDOT:PSS solar cell is around 2.64 with a fluctuation of 0.01 in the temperature of 240-290 K. Note that the value of ideality factor is not obviously changed with temperature, and meanwhile the Eq. (6) can be further written as


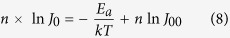


Therefore, the activation energy barrier *E*_*a*_ responsible for current rectification can be determined as 1.12 eV from a plot of n × ln(*J*_*0*_) *versus l/T*, seeing Fig. 11(b). This value fits well with the band gap of the silicon in this temperature range, suggesting the temperature dependent rectifying characteristics originate from the thermally generated carriers in silicon, and that the silicon is the light-absorbing/carrier generating active site in the LF-SiNWs/PEDOT:PSS solar cells. Such a methodology to extract the band gap of light absorbing materials has been extensively used in investigating various heterojunction solar cells^28^,^29^. This result suggests that the LF-SiNWs/PEDOT:PSS solar cell indeed works as a p-n homojunction device and the PEDOT:PSS film only functions as the electrodes here.

## Conclusions

In summary, we have prepared the uniform SiNWs with various diameters for the fabrication of SiNWs/PEDOT:PSS solar cells. It is found that the LF-SiNWs/PEDOT:PSS solar cell has the same *V*_*oc*_ as the Planar-Si/PEDOT:PSS one, the same *J*_*sc*_ as the LR-SiNWs/PEDOT:PSS one. Meanwhile, the FF of LF-SiNWs/PEDOT:PSS solar cell is much larger than those of planar-Si/PEDOT:PSS and LR-SiNWs/PEDOT:PSS ones. Therefore, a highest PCE of 13.11% can be obtained from the LF-SiNWs/PEDOT:PSS solar cell. The high built-in potential barrier at the LF-SiNWs/PEDOT:PSS interface can yield a strong inversion layer near the silicon surface, which effectively suppresses the carrier recombination, reduces and reduce the leakage current of solar cell, and meanwhile transfers the LF-SiNWs/PEDOT:PSS device into a p-n junction. These results are of significance for fabricating high efficiency silicon/PEDOT:PSS hybrid solar cells.

## Additional Information

**How to cite this article**: Yu, X. *et al.* High Efficiency Organic/Silicon-Nanowire Hybrid Solar Cells: Significance of Strong Inversion Layer. *Sci. Rep.* **5**, 17371; doi: 10.1038/srep17371 (2015).

## Figures and Tables

**Table 1:** Electronic output characteristics of the hybrid planar-Si/PEDOT:PSS, LR-SiNWs/PEDOT:PSS and LF-SiNWs/PEDOT:PSS solar cells.

	J_sc_ (mA/cm^2^)	V_oc_ (V)	FF	R_s_ (Ω cm^2^)	PCE (%)
Planar-Si	27.60	0.608	0.66	7.04	11.05
LR-SiNWs	30.35	0.574	0.53	13.24	10.17
LF-SiNWs	30.42	0.614	0.70	5.30	13.11
